# Inhibitory Effects of Dopamine Receptor D_1_ Agonist on Mammary Tumor and Bone Metastasis

**DOI:** 10.1038/srep45686

**Published:** 2017-04-04

**Authors:** Kazumasa Minami, Shengzhi Liu, Yang Liu, Andy Chen, Qiaoqiao Wan, Sungsoo Na, Bai-Yan Li, Nariaki Matsuura, Masahiko Koizumi, Yukun Yin, Liangying Gan, Aihua Xu, Jiliang Li, Harikrishna Nakshatri, Hiroki Yokota

**Affiliations:** 1 Department of Biomedical Engineering, Indiana University Purdue University Indianapolis, Indianapolis, IN 46202, USA; 2 Department of Medical Physics & Engineering Osaka University Graduate School of Medicine Suita, Osaka 565-0871, Japan; 3 Department of Pharmacology, School of Pharmacy, Harbin Medical University, Harbin 150081, China; 4 Weldon School of Biomedical Engineering, Purdue University, West Lafayette, IN 47907, USA; 5 Osaka Medical Center for Cancer and Cardiovascular Diseases, Osaka 537-8511, Japan; 6 Department of Biology, Indiana University Purdue University Indianapolis, Indianapolis, IN 46202, USA; 7 Department of Surgery, Simon Cancer Research Center, Indiana University School of Medicine, Indianapolis, IN 46202, USA

## Abstract

Dopaminergic signaling plays a critical role in the nervous system, but little is known about its potential role in breast cancer and bone metabolism. A screening of ~1,000 biologically active compounds revealed that a selective agonist of dopamine receptor D1 (DRD1), A77636, inhibited proliferation of 4T1.2 mammary tumor cells as well as MDA-MB-231 breast cancer cells. Herein, we examined the effect of A77636 on bone quality using a mouse model of bone metastasis from mammary tumor. A77636 inhibited migration of cancer cells in a DRD1-dependent fashion and suppressed development of bone-resorbing osteoclasts by downregulating NFATc1 through the elevation of phosphorylation of eIF2α. In the mouse model of bone metastasis, A77636 reduced osteolytic lesions and prevented mechanical weakening of the femur and tibia. Collectively, we expect that dopaminergic signaling might provide a novel therapeutic target for breast cancer and bone metastasis.

Dopaminergic signaling plays an important role in the central nervous system as a regulator of motivation, pleasure, cognition, memory, learning, and motor control^1^. Anomalies in dopaminergic signaling are linked to neurological and psychiatric disorders such as Parkinson’s disease, schizophrenia, and bipolar disorder^2^. Besides the central nervous system, dopamine exerts distinctive functions in various organs. Dopamine acts as a vasodilator of blood vessels, a stimulator of urine in the kidney, and a suppressor of insulin production in the pancreas^3^,^4^. There are five known dopamine receptors (D_1_–D_5_), which can be divided into D_1_-like family receptors and D_2_-like family receptors^5^. Activating D_1_-like family receptors induces adenylyl cyclase and increases the intracellular concentration of cyclic adenosine monophosphate (cAMP), while activation of D_2_-like family receptors exerts the opposite function^6^,^7^. To our knowledge, little is known about the role of dopaminergic signaling in bone metastasis from breast cancer.

One out of eight women in the U.S. suffers breast cancer in her lifetime, and a primary cause of death from breast cancer is not from primary tumors but from secondary tumors through metastasis^8^. Bone is a frequent site of metastasis, and developing an effective treatment for bone metastasis without inducing life-threatening toxicities is an urgent need^9^. In an effort to identify a new drug target for treatment of breast cancer and bone metastasis, this study conducted a screening of drug candidates from a chemical library that consisted of 1,120 biologically active compounds. From a short list of candidates in this screening, an agonist of dopamine receptor D_1_ (DRD1), A77636^10^, was selected for further validation.

To assess the therapeutic potential of A77636, we first examined its ability to inhibit proliferation and migration of mammary tumor cells and its ability to prevent bone resorption. In order to examine the involvement of dopaminergic signaling, silencing by RNA interference was conducted using siRNA specific to DRD1. The potential linkage between regulation of DRD1 and Rac1 GTPase in cell migration was also evaluated using live cell imaging with a FRET (fluorescence resonance energy transfer) technique^11^. To test the effects of A77636 *in vivo*, 4T1.2 mammary tumor cancer cells were injected into the mammary pad to examine tumor growth, as well as an external iliac artery for evaluating bone metastasis in the hindlimb.

## Results

### Suppression of tumor growth by A77636

**Figure 1:**
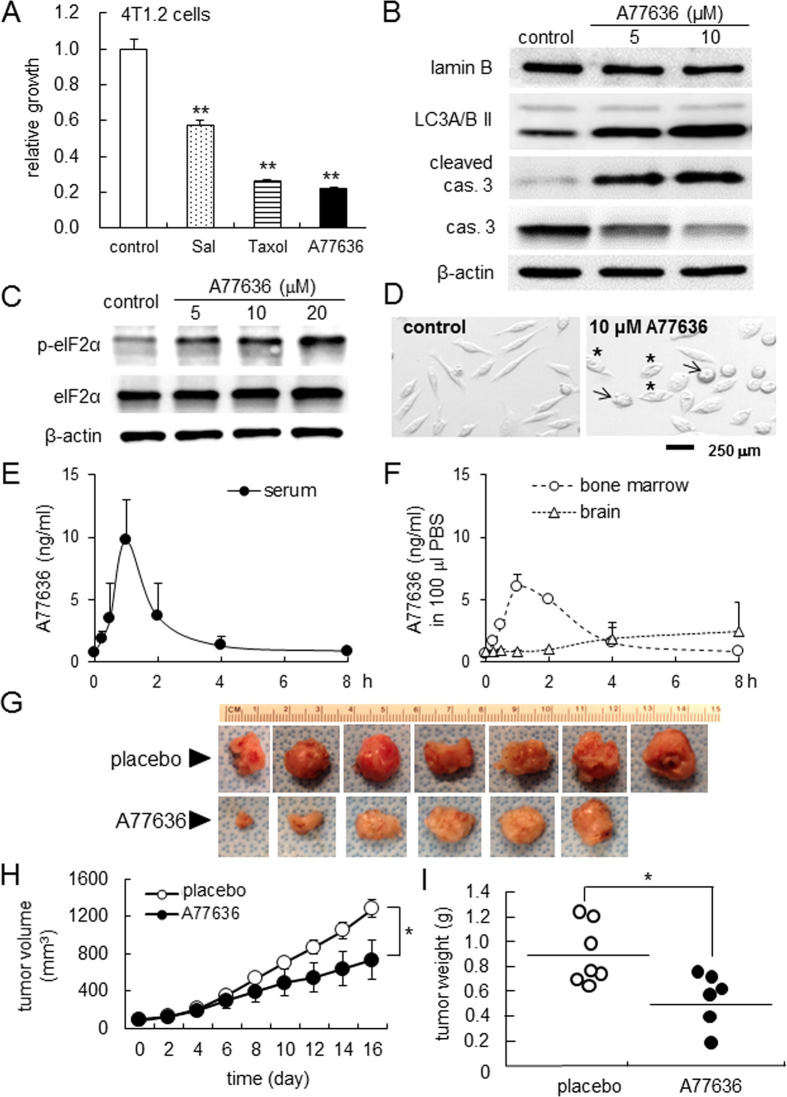
Suppression of tumor growth by A77636. The single and double asterisks indicate *p* < 0.05 and p < 0.01, respectively. (**A**) Reduced growth of 4T1.2 mammary tumor cells by 10 μM of Sal (salubrinal), Taxol, and A77636. Salubrinal is an inhibitor of PP1 phosphatase that de-phosphorylates eIF2α. (**B**) Expression of lamin B as a marker for integrity of nuclear envelopes, microtubule associated protein light chain 3 A/B II (LC3A/B II) as a marker for autophagy, and cleaved and uncleaved caspase 3 as a marker for apoptosis, in response to 5 and 10 μM A77636 in 4T1.2 cells. (**C**) Upregulation of eIF2α phosphorylation by A77636 in 4T1.2 cells. (**D**) Images of normal (control) and damaged (A77636-treated) 4T1.2 cells. The arrow and asterisk indicate apoptotic and autophagy cells, respectively. (**E**) Concentration of A77636 in the serum in response to i.p. injection of A77636 at a dose of 2 mg/kg. (**F**) Detection of A77636 in the bone marrow (femur), and brain (frontal cortex, ~60 mg) in response to i.p. injection of A77636 at a dose of 2 mg/kg. The femoral bone marrow and frontal cortex were dissolved in 100 μl PBS, and the concentration was measured. (**G**) Mammary tumors at the injected site of mammary fat pad for the placebo (N = 7) and A77636-treated mice (N = 6). (**H**,**I**) Volume (mm^3^) and weight (g) of mammary tumors.

Out of 1,120 biologically active compounds, we focused on A77636 as a potential agent that inhibits growth of breast cancer cells (MDA-MB-231 cells and MDA-MB-436 cells) without significantly affecting growth of normal epithelial cells (MCF10A cells) and bone-forming osteoblasts (MC3T3 cells) (Suppl. Fig. 1A–D). In 4T1.2 cells, 10 μM A77636 reduced their proliferation by elevating an apoptotic marker, cleaved caspase 3, and an autophagy marker, LC3A/B II, as well as the phosphorylation level of eukaryotic translation initiation factor 2α (p-eIF2α) (Fig. 1A–D). Salubrinal, Fenoldopam, and Taxol were employed as control agents, which were the modulator of p-eIF2α, DRD1 agonist, and a chemotherapeutic agent for breast cancer, respectively *(Suppl. Fig. 2A–C). The estimated EC50% for blocking 50% cell proliferation in 4T1.2 cells was estimated as 2.0 μM (Suppl. Fig. 2D). In response to 2 μM A77636, the level of p-eIF2α was elevated, and the expression of DRD1 was elevated (Suppl. Fig. 1E, Suppl. Fig. 2E).

Intraperitoneal injection of A77636 to mice at a dose of 2 mg/kg made its serum concentration ~10 ng/ml, 1 h after injection with a half-life of ~1 h (Fig. 1E). In the mouse model of mammary tumor, in which 4T1.2 cells were injected into the mammary fat pad, daily intraperitoneal injections of A77636 (2 mg/kg) for 2 weeks significantly reduced the volume and weight of the tumor at the injection site (Fig. 1G–I). Furthermore, the bone mineral density of the femur and humerus was significantly elevated in the A77636-treated group (Suppl. Fig. 2F).

### Inhibitory effects of A77636 on cellular motility

**Figure 2:**
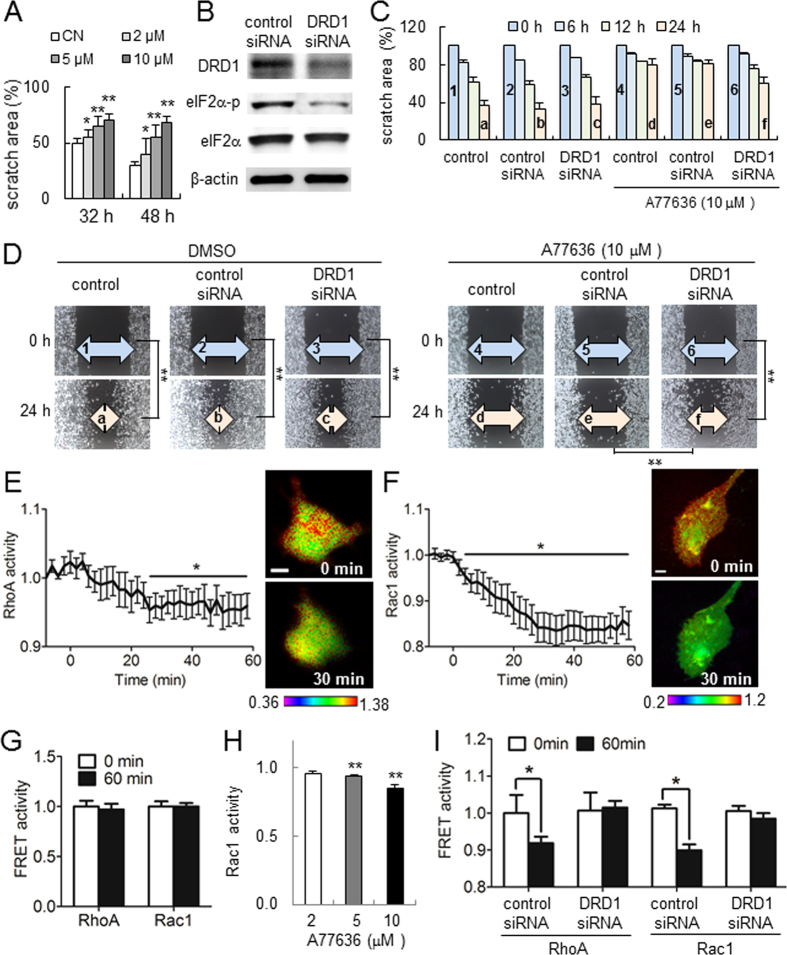
Inhibitory effects of A77636 on the migration of MD231 cells. The single and double asterisks indicate *p* < 0.05 and *p* < 0.01, respectively. (**A**) Dose-dependent suppression of cellular migration in response to 2, 5, and 10 μM A77636. Of note, CN = control. (**B**) Partial silencing of dopamine receptor D1 (DRD1) with DRD1 siRNA. (**C**,**D**) Wounded area with and without DRD1 siRNA in the presence and absence of 10 μM A77636. The double asterisk indicates *p* < 0.01. (**E**,**F**) FRET-based activities of RhoA and Rac1 GTPases, respectively, in response to 10 μM A77636. (**G**) Stationary FRET-based baseline activities of RhoA and Rac1 GTPases in the absence of A77636. (**H**) Rac1 activity after 1 h treatment with 1, 5, and 10 μM A77636. (**I**) FRET-based RhoA and Rac1 activities in the presence and absence of DRD1 siRNA in response to 10 μM A77636.

In the wound healing scratch assay, A77636 reduced cellular motility of MDA-MB-231 cells in a dose-dependent manner at 2, 5, and 10 μM (Fig. 2A). However, a partial silencing of DRD1 with siRNA specific to DRD1 abolished A77636′s reduction in cellular motility (Fig. 2B–D). Of note, the result in the scratch assay is affected not only by cell migration but also by cell proliferation. Live cell imaging for determining FRET-based activity revealed that compared to a placebo control, 10 μM A77636 significantly decreased the activity of RhoA and Rac1 GTPases (Fig. 2E–G). Furthermore, A77636 reduced Rac1 activity in a dose dependent manner (Fig. 2H). Consistent with the result in cellular motility, RNA interference with DRD1 siRNA suppressed A77636-driven reduction in FRET-based activities of RhoA and Rac1 (Fig. 2I).

### Effects of A77636 on bone-resorbing osteoclasts and bone-forming osteoblasts

**Figure 3:**
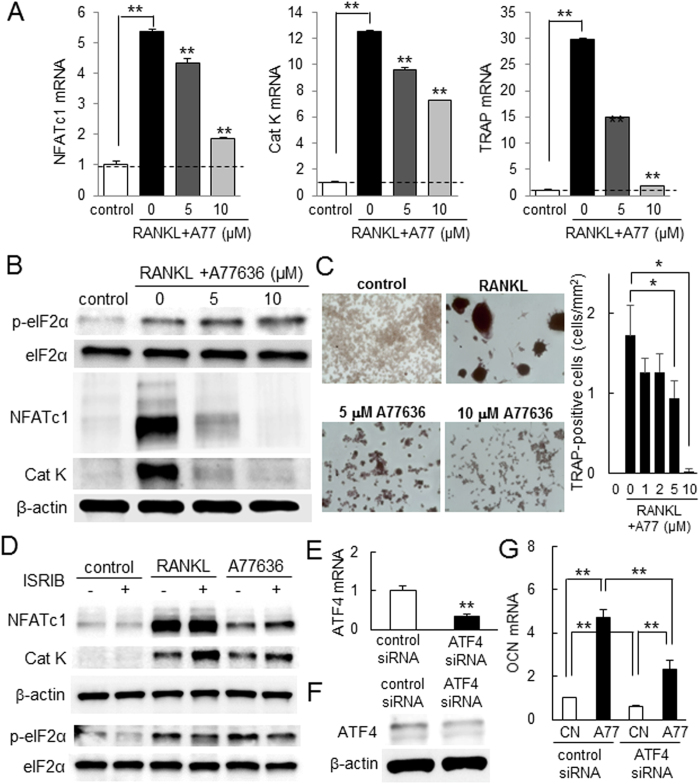
Inhibitory effects of A77636 on development of RAW267.4 pre-osteoclasts. The double asterisk indicates *p* < 0.05. Of note, A77 = A77636, and CN = control. (**A**) Decrease in the relative mRNA abundance of NFATc1, Cathepsin K, and TRAP in response to 5 and 10 μM A77363 in RANKL stimulated cells. (**B**) Downregulation of NFATc1, Cathepsin K, and TRAP proteins in response to 5 and 10 μM A77363 in RANKL stimulated cells. (**C**) Suppression of TRAP-positive multinucleated cells by 1–10 μM A77636. The number of TRAP-positive cells with 3 or more nuclei was counted. (**D**) Suppressive effects of ISRIB on A77636-driven downregulation of NFATc1 and Cat K. (**E**,**F**) Partial reduction of ATF4 mRNA and ATF4 protein, respectively, with siRNA specific to ATF4. (**G**) ATF4-mediated upregulation of osteocalcin (OCN) mRNA in response to 10 μM A77636.

In response to RANKL stimulation in RAW264.7 pre-osteoclasts, administration of A77636 downregulated the mRNA and protein levels of NFATc1, cathepsin K, and TRAP in a dose-dependent fashion (Fig. 3A and B). Furthermore, A77363 reduced the number of TRAP-positive multi-nucleated osteoclasts (Fig. 3C). In response to ISRIB that inhibits phosphorylation of eIF2α, A77636-driven suppression of NFATc1 and Cathepsin K was significantly suppressed (Fig. 3D). Fenoldopam is a partial agonist of DRD1, while Pimozide is an antagonist of DRD2. In response to 5 and 10 μM Fenoldopam, the level of p-eIF2α was elevated and that of Cathepsin K was downregulated, although the efficacy of A77636 was significantly stronger than that of Fenoldopam (Suppl. Fig. 3A–C). Administration of 10 μM Pimozide significantly elevated p-eIF2α and downregulated NFATc1 (Suppl. Fig. 3D and E).

In addition, A77636 presented stimulatory effects on development of bone-forming osteoblasts without significantly altering cell proliferation (Suppl. Fig. 1A). In response to 10 μM A77636, the levels of p-eIF2α, ATF4, and alkaline phosphatase (ALP) were elevated in MC3T3 osteoblast-like cells (Suppl. Fig. 4B–D). Furthermore, the mRNA level of osteocalcin (OCN) was elevated in a dose dependent manner in response to 2 and 10 μM A77636 for 6 days (Suppl. Fig. 4E). A partial silencing of ATF4 by RNA interference suppressed A77636-driven elevation of osteocalcin mRNA (Fig. 3E–G).

### Protection of bone loss by A77636 in the mouse model of bone metastasis

**Figure 4:**
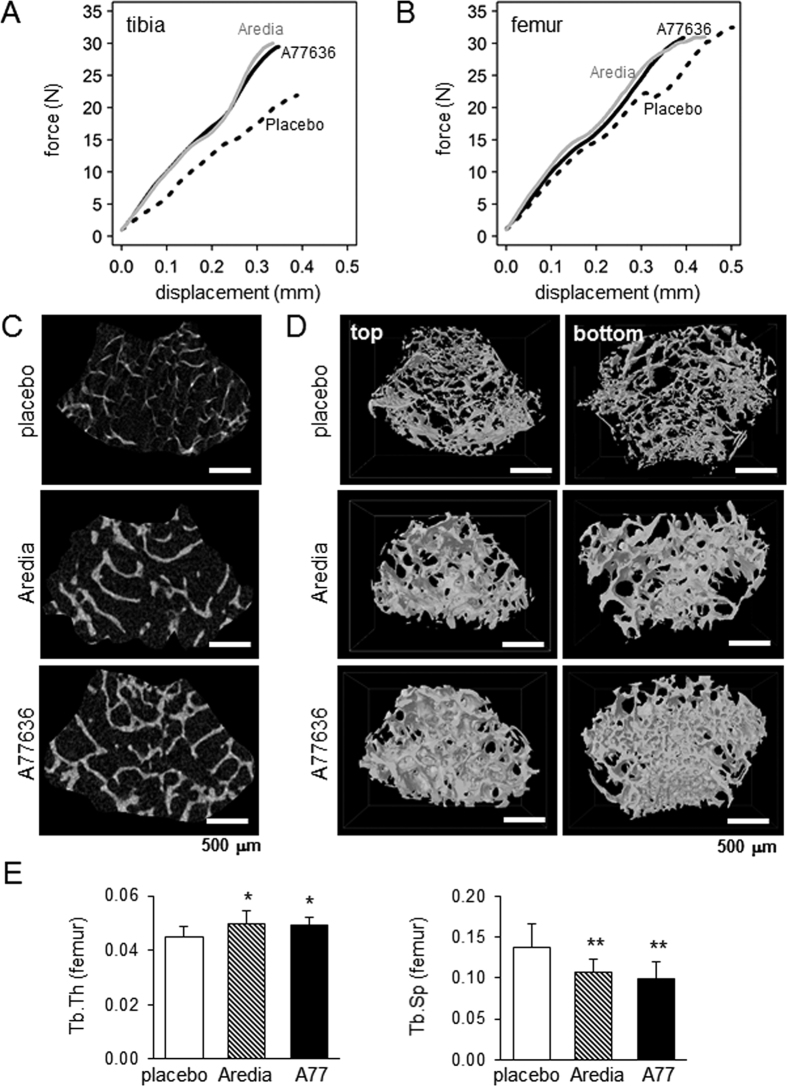
Protection of bone loss by A77636 in the mouse model of bone metastasis. The single and double asterisks indicate *p* < 0.05 and p < 0.01, respectively. (**A**,**B**) Force-displacement relationship for the tibia and femur, respectively. Aredia is a bisphosphonate control. (**C**,**D**) μCT images for the placebo, Aredia, and A77636 groups as a 2-dimensional section and 3-dimensional reconstructed structure, respectively. (**E**) Two parameters for trabecular bone in the distal femur. Of note, A77 = A77636, Tb. Th = trabecular thickness, and Tb. Sp = trabecular spacing.

In the mouse model of bone metastasis, 4T1.2 cells were injected into the right iliac artery and mechanical and structural changes in the right hindlimb were investigated among three groups (placebo, Aredia, and A77636). Aredia is a bisphosphonate and used as a positive control for blocking bone resorption. In the force-displacement relationship for the tibia and femur, Aredia and A77636 groups presented higher stiffness as well as maximum force than the placebo group (Fig. 4A and B; Suppl. Fig. 5A and B). Two-dimensional sectioning and 3-dimensional reconstruction from μCT images of the distal femur (0.5 to 1 mm proximal to the growth plate) revealed that A77636 generated a denser trabecular structure than the placebo (Fig. 4C and D). Furthermore, compared to placebo, administration of A77636 yielded a higher trabecular thickness, with lower trabecular spacing in the femur (Fig. 4E; Suppl. Fig. 5C for the tibia). Histological analysis of osteolytic lesions in the distal femur revealed several broken pieces of cortical bone in the placebo group as well as a tumor cell-invaded area in bone marrow of Aredia group (Suppl. Fig. 6). Compared to placebo, the bone degradation ratio and tumor area ratio were significantly smaller in A77636-treated group. A clinical score based on X-ray images of hindlimbs indicated that Aredia and A77636 groups received a significantly better score than the placebo group (Suppl. Fig. 5D).

### Remodeling of trabecular bone in the distal femur

**Figure 5:**
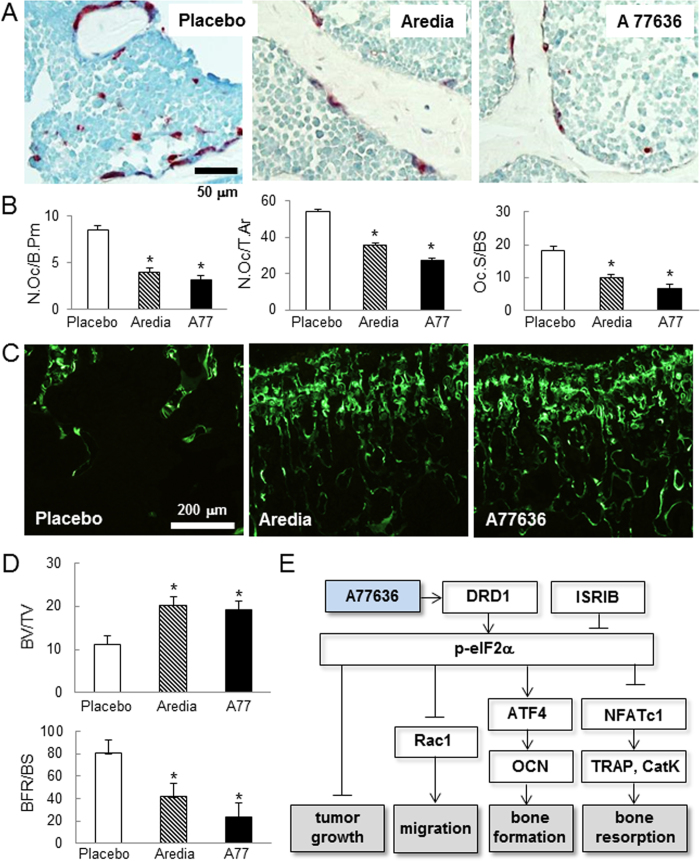
Remodeling of trabecular bone in the distal femur in the mouse model of bone metastasis. Three mouse groups are placebo, Aredia, and A77 (A77636). The single asterisk indicates *p* < 0.05. (**A**) TRAP staining. (**B**) Three osteoclast parameters. Of note, N.Oc/B.Pm = number of osteoclasts normalized by bone perimeter, N.Oc/T.Ar = number of osteoclasts normalized by tissue area, and Oc.S/BS = osteoclast surface normalized by bone surface. (**C**) Calcein-stained trabecular bone in the distal femur. (**D**) Bone volume normalized by tissue volume (BV/TV) and bone formation rate normalized by bone surface (BFR/BS). (**E**) DRD1 and eIF2α mediated mechanism of A77636′action on tumor growth, migration, and bone formation and resorption.

Histological analysis based on TRAP staining revealed that A77636 group provided the most effective bone protection with the smallest values in three osteoclast-related parameters (N.Oc/B.Pm = number of osteoclasts normalized by bone perimeter; N.Oc/T.Ar = number of osteoclasts normalized by tissue area; and Oc.S/BS = osteoclast surface normalized by bone surface) (Fig. 5A and B). Furthermore, histological images with calcein staining showed that bone volume normalized by tissue volume (BV/TV) was higher in A77636 group than that in the placebo (Fig. 5C and D). However, the bone formation rate normalized by bone surface (BFR/BS) was the highest in the placebo and the lowest in A77636 group, since bone surface (BS) for normalizing BFR is the smallest in the placebo and the largest in A77636 group (Fig. 5D). A77636 also elevated bone mineral density of the upper limb (ulna and humerus) (Suppl. Fig. 5E).

## Discussion

We present in this study an agonist of DRD1, A77636, upregulated the phosphorylation of eIF2α and inhibited proliferation and motility of breast cancer cells as well as bone loss and weakening. Through upregulation of p-eIF2α, A77636 suppressed cellular proliferation and migration of 4T1.2 mammary tumor cells and MDA-MB-231 breast cancer cells. It also enhanced development of bone-forming osteoblasts, and inhibited that of bone-resorbing osteoclasts. In the mouse model of mammary tumor, A77636 significantly reduced tumor volume and weight in the mammary pad. In the mouse model of bone metastasis, A77636 reduced the invasion of tumor cells in the bone marrow cavity as well as the osteolytic lesions. Collectively, the results from *in vitro* and *in vivo* data herein indicate that A77636 is capable of protecting bone from mammary tumor derived bone metastasis.

In 4T1.2 mammary tumor cells, administration of A77636 elevated the level of cleaved caspase 3 as a marker of apoptosis^12^, as well as the level of LC3-phosphatidylethanolamine conjugate (LC3II) as an autophagosome marker^13^. We have previously reported that salubrinal is capable of inducing cellular death of 4T1 cells as well as MDA-MB-231 cells by elevating p-eIF2α^14^. A77636 also elevated p-eIF2α, and A77636-driven increase in cleaved caspase 3 was suppressed by ISRIB, the selective inhibitor of eIF2α phosphorylation. The primary role of autophagy is cytoprotection from various cellular stresses, but it also leads to cell death in the presence of intense and/or prolonged cellular stress^15^. The results in this study are consistent with the notion that A77636 induces apoptotic cell death and autophagy-linked growth arrest via dopaminergic signaling.

In FRET-based live imaging of MDA-MB-231 cells, A77636 significantly reduced activity of Rac1 GTPase, as well as that of RhoA GTPase to a minor degree. Rac1 GTPase regulates various cellular processes, such as cell cycle, motility, invasion, and cell-cell adhesion^16^, and plays a critical role in the development and metastasis of various cancers, including breast cancer^17^. Salubrinal is also reported to inhibit Rac1 activity via eIF2α-mediated signaling^18^. In SW1353 chondrosarcoma cells, it is reported that Rac1 GTPase was suppressed by the elevation of p-eIF2α^19^. Further analysis is necessary to examine whether A77636′s action on Rac1 GTPase and/or RhoA GTPase may regulate matrix metalloproteinases such as MMP2 and MMP9 that may drive migration of breast cancer cells^20^.

Regarding development of bone-forming osteoblasts and bone-resorbing osteoclasts, we have previously shown that the upregulation of p-eIF2α by salubrinal stimulates development of osteoblasts and suppresses development of osteoclasts. Salubrinal is known to bind protein phosphatase 1 alpha subunit (PP1α) and reduce stress to the endoplasmic reticulum^21^. Since PP1 is a selective phosphatase of eIF2α, salubrinal’s inhibition of de-phosphorylation of eIF2α contributes to elevation of p-eIF2α. In dopaminergic signaling, the activation of DRD1 stimulates dopamine- and cAMP-regulated phosphoprotein, 32 kDa (DARPP32), which is a potent inhibitor of PP1. Consistent with salubrinal’s role in eIF2α-mediated bone regulation, A77636′s upregulation of p-eIF2α also led to ATF4-mediated development of osteoblasts. More importantly, A77636 acted as a potent inhibitor of NFATc1 and suppressed RANKL-driven development of osteoclasts.

For the treatment of bone metastasis, the current available therapies include administration of zoledronic acid (Zometa) as a bisphosphonate, and denosumab (Xgeva) as a neutralizing antibody against RANKL^22^,^23^. Although the most commonly used drug is a bisphosphonate that inhibits bone-resorbing osteoclasts and suppresses osteolytic lesions, it does not stimulate bone formation. Furthermore, its high dosage presents detrimental effects such as joint inflammation and avascular osteonecrosis of the jaw^24^. In a randomized clinical trial, Denosumab presents fewer bone-related complications. However, Denosumab’s effects on tumor growth in breast cancer have yet to be elucidated. This study showed that A77636 is capable of stimulating bone formation through upregulating ATF4, as well as inhibiting bone resorption by inhibiting NFATc1. Furthermore, it is able to reduce the proliferation and migration of 4T1.2 mammary tumor cells and MDA-MB-231 breast cancer cells.

In this study, we employed 4T1.2 cells and MDA-MB-231 cells that are both triple negative (i.e., negative for estrogen receptor, progesterone receptor, and human epidermal growth factor receptor 2). Since bone metastasis is more often caused by ER positive breast cancer, it is important to evaluate A77636’s effects on ER positive cells. For mouse models of bone metastasis, tumor cells are often inoculated into the left ventricle of the heart^25^. In this method, cancer cells tend to spread to the whole body. Inoculating cancer cells into the artery of the tail is also known to induce lung metastasis. We chose a method of inoculating into the iliac artery, which had a high probability of inducing bone metastasis with a low probability of metastasis to lung^26^. In our animal study, we observed bone metastasis in the right hindlimb since we inoculated the right iliac artery. No significant difference was observed in the lung weight between the placebo and A77636-treated groups (data not shown).

It is reported that a partial agonist of DRD1-like receptors, Fenoldopam, causes tumor shrinkage in the mouse xenograft model through the cGMP/protein kinase G (PKG) pathway^27^. We also observed reduction in cell proliferation in 4T1.2 cells (Suppl. Fig. 2A), although the reduction with Fenoldopam was smaller than that with A77636. While Fenoldopam suppressed RANKL-driven upregulation of Cathepsin K in pre-osteoclasts, it did not significantly alter the expression of NFATc1. The current study focused on DRD1/cAMP/DARPP32 pathway and its linkage to eIF2α phosphorylation, which led to the regulation of bone-forming osteoblasts and bone-resorbing osteoclasts. To further evaluate the role of dopaminergic signaling, we employed Pimozide, an antagonist of DRD2-like receptors. Because of the differential roles of DRD1-like and DRD2-like receptors in dopaminergic signaling, DRD2 antagonists, such as Pimozide^28^, are expected to induce responses similar to those by DRD1 agonists, such as A77636 and Fenoldopam. In RAW264.7 cells, Pimozide elevated p-eIF2α and suppressed RANKL-driven expression of NFATc1 (Suppl. Fig. 3D and E). Taken together, there are variations in affinity and selectivity among dopaminergic agonists and antagonists, and further analysis is recommended to identify more potent agents to inhibit bone resorption.

DRD1 agonists such as Levodopa are frequently administered to the patients with Parkinson’s disease, who generally present lower bone mineral density^29^. It is recommended to examine A77636′s effect on bone quality in the patients with Parkinson’s disease. In conclusion, we demonstrate that an inhibitory agent of de-phosphorylation of eIF2α through agonizing DRD1 potentially offers a novel therapeutic option for attenuating malignant phenotypes of triple negative breast cancer cells, as well as reducing osteolytic lesions from bone metastasis. Dopaminergic signaling may provide a novel therapeutic target for breast cancer and bone metastasis.

## Materials and Methods

### Cell culture

4T1.2 mouse mammary tumor cells [obtained from Dr. R. Anderson at Peter MacCallum Cancer Institute, Melbourne, Australia^30^]; and MDA-MB-231 human breast cancer cells [American Type Culture Collection (ATCC), Manassas, VA, USA^31^]; were cultured in DMEM. MDA-MB-436 human breast cancer cells ATCC^32^; were grown in MEM, and MCF10A human breast epithelial cells^33^ were cultured in DMEM-F12 with 1 mg/ml hydrocortisone and 2 μg/μl Epidermal Growth Factor (EGF). RAW264.7 pre-osteoclast cells (ATCC) and MC3T3 osteoblast-like cells (Sigma-Aldrich, St. Louis, MO, USA) were grown in αMEM. The culture media contained 10% fetal bovine serum (FBS) and antibiotics (50 units/ml penicillin, and 50 μg/ml streptomycin; Life Technologies, Grand Island, NY, USA). Cells were maintained at 37 °C and 5% CO_2_ in a humidified incubator.

A77636 was employed as a selective DRD1-like receptor agonist, while salubrinal and ISBIB were used as the stimulator and inhibitor of eIF2α phosphorylation, respectively. It is reported that EC50 of A77636 is 1.1 nM to DRD1 and above 10 μM to DRD2 34. Fenoldopam was an FDA approved agent for partially agonizing DRD1-like receptors, and cGMP analog and KT5823 were used as a stimulator and inhibitor of protein kinase G. Pimozide was employed as an antagonist of DRD2-like receptor. All agents were purchased from R&D Systems unless otherwise specified.

### Pharmacokinetic analysis

A77636′s concentration (in ng/ml) in the serum, bone marrow, and brain was determined using a liquid chromatography/mass spectrometry based assay. Using BALB/c mice (female, ~8 weeks old), A77636 at 2 mg/kg was injected via i.p. The blood, bone marrow from a pair of femora, and the frontal cortex (~60 mg) were harvested at 0 min, 15 min, 30 min, 1 h, 2 h, 4 h, and 8 h after injection. The extract from the bone marrow from a single femur as well as the frontal cortex was suspended in PBS and the final volume was adjusted to 100 μl.

### Two-dimensional motility assay

To evaluate 2-dimensional motility, a wound healing scratch motility assay was carried out as described previously^35^. In brief, cells were plated in 12-well plates or 6-cm dishes and on the next day, scratching was performed using a plastic tip. The areas newly occupied with cells in the scratched zone were determined 24 h after scratching using images obtained by a microscope, which were scanned with Adobe Photoshop (CS2, Adobe Systems, San Jose, CA, USA) and quantified with ImageJ.

### Fluorescence resonance energy transfer (FRET)

To visualize activities of RhoA and Rac1 GTPases in response to 10 μM A77636, FRET imaging was conducted using cyan fluorescent protein (CFP)-yellow fluorescent protein (YFP) biosensors^36^. The filter sets (Semrock) were chosen for CFP excitation at 438 ± 24 nm (center wavelength ± bandwidth), CFP emission at 483 ± 32 nm, and YFP emission at 542 ± 27 nm. Time-lapse images were acquired at an interval of 5 min using a fluorescence microscope (Nikon, Tokyo, Japan). The activity levels were determined by computing an emission ratio of YFP/CFP for individual cells using NIS-Elements software (Nikon).

### Osteoclast differentiation assay

Using RAW264.7 pre-osteoclast cells^37^, the osteoclast differentiation assay was conducted in 96-well plates with 20 ng/ml of RANKL in the presence and absence of A77636. During 6-day experiments, the culture medium was exchanged once on day 4. Adherent cells were fixed and stained with a tartrate resistant acid phosphate (TRAP)-staining kit according to the manufacturer’s instructions^38^. TRAP-positive multinucleated cells (>3 nuclei) were identified as mature osteoclasts.

### Real-time qPCR

Total RNA was extracted using an RNeasy Plus mini kit (Qiagen, Germantown, MD, USA) and reverse transcription was conducted with high capacity cDNA reverse transcription kits (Applied Biosystems, Carlsbad, CA, USA). Real-time qPCR was performed using Power SYBR green PCR master mix kits (Applied Biosystems) with PCR primers listed in Table 1.

### Western blot analysis

Cells were lysed in a radio-immunoprecipitation assay (RIPA) buffer with protease inhibitors (Santa Cruz Biotechnology, Santa Cruz, CA, USA) and phosphatase inhibitors (Calbiochem, Billerica, MA, USA). Isolated proteins were fractionated using 10–15% SDS gels and electro-transferred to polyvinylidene difluoride membranes (Millipore, Billerica, MA, USA). The membrane was incubated for 1 h with primary antibodies followed by 45 min incubation with secondary antibodies conjugated with horseradish peroxidase (Cell Signaling, Danvers, MA, USA). We used antibodies against ATF4, caspase 3, cathepsin K, DRD1, eIF2α, p-eIF2α, lamin B, LC3A/B II, NFATc1 (Cell Signaling), and β-actin (Sigma). Protein levels were assayed using a SuperSignal west femto maximum sensitivity substrate (Thermo Scientific, Waltham, MA, USA), and signal intensities were quantified with a luminescent image analyzer (LAS-3000, Fuji Film, Tokyo, Japan).

### Knockdown of DRD1 and ATF4 by siRNA

Cells were treated with siRNA specific to DRD1 and ATF4 (Life Technologies). Selected target sequences for knockdown of DRD1 and ATF4 were: DRD1, 5′-AUG AGU ACA GAC AAG GUC CAT-3′, and ATF4, 5′-GCU GCU UAC AUU ACU CUA A-3′. As a nonspecific control, a negative siRNA (Silencer Select #1, Life Technologies) was used. Cells were transiently transfected with siRNA in Opti-MEM I medium with Lipofectamine RNAiMAX (Life Technologies). Six hours later, the medium was replaced by regular culture medium. The efficiency of silencing was assessed with immunoblotting 48 h after transfection.

### Animal models

The experimental procedures were approved by the Indiana University Animal Care and Use Committee and were in compliance with the Guiding Principles in the Care and Use of Animals endorsed by the American Physiological Society. Five mice were housed per cage, and fed with mouse chow and water *ad libitum*. In the mouse model of mammary tumor^39^, 16 BALB/c female mice (~6 weeks, Harlan Laboratories) were used. Mice received subcutaneous injection of 4T1.2 cells (5.0 × 10^5^ cells in 50 μl PBS) to the mammary fat pad on day 1. A77636 (1 mg/kg body weight) was administered subcutaneously into the area of cell injection every day, while the placebo control animals received a vehicle. The animals were sacrificed on day 18 and the volume and weight of tumors were determined. The tumor volume was calculated as (long diameter) × (short diameter)^2^/2. In the mouse model of bone metastasis^26^, 20 BALB/c female mice received injection of 4T1.2 cells (1.0 × 10^5^ cells in 50 μl PBS) to the right iliac artery. A77636 was daily administered as i.p. injection at 2 mg/kg body weight, while Aredia was weekly given as a bisphosphonate control at 0.5 mg/kg. The animals were sacrificed on day 17. A whole body X-ray was taken, and the tibia and femur were harvested for mechanical testing, μCT imaging, and histology.

### Mechanical testing

The tibia and femur samples were tested to failure by four-point bending using a voltage-regulated mechanical loading device (Electro Force 3100, Bose, Inc.), with a loading span of 2.3 mm and a support span of 7 mm^40^. The load was applied to the longitudinal center. After preloading to 0.5 N, the bone was loaded monotonically at 0.005 mm/s until failure. Load and displacement were recorded and used to calculate stiffness and maximum force.

### μCT imaging

Micro-computed tomography was performed using Skyscan 1172 (Bruker-MicroCT, Kontich Belgium)^41^. The harvested bone samples were wrapped in parafilm to maintain hydration and placed in a plastic tube and oriented vertically. Scans were performed at pixel size 8.99 μm. Using manufacturer-provided software, the images were reconstructed (nRecon v1.6.9.18).

### Histology

Calcein fluorescent dyes were injected 1 and 2 weeks before sacrifice to calculate bone formation rate (BFR), mineralizing surface/bone surface (MS/BS), and mineral apposition rate (MAR)^42^. The bone specimens were fixed in 10% neutral buffered formalin for a minimum of 24 h, dehydrated through a series of graded alcohols, cleared in xylene, and embedded in liquid methyl methacrylate. Once polymerized, the blocks were sectioned with a rotary microtome equipped with a tungsten-carbide knife. For dynamic histomorphometry, sections were left un-plasticized and cover-slipped using a Eukitt mounting agent. For static histomorphometry, sections were deplasticized in acetone and differentially stained by a tartrate-acid resistant acid phosphatase (TRAP) stain kit (Sigma). Plastic embedded sections of the right femur were analyzed with an Olympus BX53 light/fluorescent microscope and Olympus DP72 camera interfaced with Osteomeasure™ software version 1.01 (OsteoMetrics Inc, Decatur GA). Images were analyzed at 200× magnification. Abbreviations were made according to Parfitt *et al*.^43^. Mice lacking one of the fluorescent labels were given a mineral appositional rate of 0.1 μm/day. This serves to avoid a MAR of zero and allows BFR calculation. Three parameters were: MS/BS = {dL.Pm + (sL.Pm/2)}/B.Pm, MAR = Ir.L.Th/(time between labeling), and BFR = MAR × (MS/BS). All regions analyzed were roughly from a 1.5 mm^2^ area, which was 0.8 mm proximal from the growth plate of the right femur, and 0.5 mm medial from cortical bone.

### Statistical analysis

The data were expressed as mean ± standard deviation. One-way analysis of variance was employed to examine statistical significance among groups, and Fisher’s protected least significant difference was conducted as a *post hoc* test for the pairwise comparisons. Statistical significance for *p* < 0.05 was assumed.

## Additional Information

**How to cite this article**: Minami, K. *et al*. Inhibitory Effects of Dopamine Receptor D1 Agonist on Mammary Tumor and Bone Metastasis. *Sci. Rep.* **7**, 45686; doi: 10.1038/srep45686 (2017).

**Publisher's note:** Springer Nature remains neutral with regard to jurisdictional claims in published maps and institutional affiliations.

## Supplementary Material

Supplementary Information

## Figures and Tables

**Table 1:** Real-time qPCR primers used in this study.

Target	Forward primer	Backward primer
ATF4	5′-TGGCGAGTGTAAGGAGCTAGAAA-3′	5′-TCTTCCCCCTTGCCTTACG-3′
Cat K	5′-CAGCTTCCCCAAGATGTGAT-3′	5′-AGCACCAACGAGAGGAGA AA-3′
NFATc1	5′-GGTGCTGTCTGGCCATAACT-3′	5′-GCGGAAAGGTGGTATCTCAA-3′
OCN	5′-CCGGGAGCAGTGTGAGCTTA-3′	5′-AGGCGGTCTTCAAGCCATACT-3′
TRAP	5′-TCCTGGCTCAAAAAGCAGTT-3′	5′-ACATAGCCCACACCGTTCTC-3′
GAPDH	5′-TGCACCACCAACTGCTTAG-3′	5′-GGATGCAGGGATGATGTTC-3′

## References

[b1] Girault J. A. & Greengard P. The neurobiology of dopamine signaling. Arch Neurol. 61, 641–644 (2004). 15148138 10.1001/archneur.61.5.641

[b2] Beaulieu J. M. & Gainetdinov R. R. The physiology, signaling, and pharmacology of dopamine receptors. Pharmacol Rev. 63, 182–217 (2011). 21303898 10.1124/pr.110.002642

[b3] Zhang M. Z. & Harris R. C. Antihypertensive mechanisms of intra-renal dopamine. Curr Opin Nephrol Hypertens. 24, 117–22 (2015). 25594544 10.1097/MNH.0000000000000104 PMC4651846

[b4] Nash A. I. Crosstalk between insulin and dopamine signaling: A basis for the metabolic effects of antipsychotic drugs. J Chem Neuroanat 16, S0891–0618 (2016). 10.1016/j.jchemneu.2016.07.010 27480675

[b5] Ott T., Jacob S. N. & Nieder A. Dopamine receptors differentially enhance rule coding in primate prefrontal cortex neurons. Neuron 84, 1317–1328 (2014). 25482027 10.1016/j.neuron.2014.11.012

[b6] Lidow M. S., Wang F., Cao Y. & Goldman-Rakic P. S. Layer V neurons bear the majority of mRNAs encoding the five distinct dopamine receptor subtypes in the primate prefrontal cortex. Synapse 28, 10–20 (1998). 9414013 10.1002/(SICI)1098-2396(199801)28:1<10::AID-SYN2>3.0.CO;2-F

[b7] Almeida J. & Mengod G. D2 and D4 dopamine receptor mRNA distribution in pyramidal neurons and GABAergic subpopulations in monkey prefrontal cortex: implications for schizophrenia treatment. Neuroscience 170, 1133–1139 (2010). 20727949 10.1016/j.neuroscience.2010.08.025

[b8] Miller K. D. . Cancer treatment and survivorship statistics, 2016. CA Cancer J Clin. 66, 271–289 (2016). 27253694 10.3322/caac.21349

[b9] Guise T. A. Breast cancer bone metastases: it’s all about the neighborhood. Cell 154, 957–959 (2013). 23993088 10.1016/j.cell.2013.08.020

[b10] Martin Y. C. The Discovery of Novel Selective D1 Dopaminergic Agonists: A-68930, A-77636, A-86929, and ABT-413. Int J Med Chem. 2011, 424535 (2011). 25954518 10.1155/2011/424535 PMC4412209

[b11] Forster T. Intermolecular energy migration and fluorescence. Ann Phys 2, 55–75 (1948).

[b12] Murad H. . Induction of G1-phase cell cycle arrest and apoptosis pathway in MDA-MB-231 human breast cancer cells by sulfated polysaccharide extracted from Laurencia papillosa. Cancer Cell Int. 16, 39 (2016). 27231438 10.1186/s12935-016-0315-4 PMC4881178

[b13] Conrad C. Weihl Monitoring Autophagy in the Treatment of Protein Aggregate Diseases: Steps Toward Identifying Autophagic Biomarkers Neurotherapeutics. 10, 383–390 (2013). 10.1007/s13311-013-0180-y PMC3701771 23408309

[b14] Hamamura K., Tanjung N. & Yokota H. Suppression of osteoclastogenesis through phosphorylation of eukaryotic translation initiation factor 2 alpha. J. Bone Miner. Metab. 31, 618–628 (2013). 23536193 10.1007/s00774-013-0450-0

[b15] Liu Y. & Levine B. Autosis and autophagic cell death: the dark side of autophagy. Cell Death Differ 22, 367–376 (2015). 25257169 10.1038/cdd.2014.143 PMC4326571

[b16] Hanna S. & El-Sibai M. Signaling networks of Rho GTPases in cell motility. Cellular Signaling. 25, 1955–1961 (2013). 10.1016/j.cellsig.2013.04.009 23669310

[b17] Bid H. K., Roberts R. D., Manchanda P. K. & Houghton P. J. RAC1: an emerging therapeutic option for targeting cancer angiogenesis and metastasis. Mol Cancer Ther. 12, 1925–1934 (2013). 24072884 10.1158/1535-7163.MCT-13-0164 PMC3823055

[b18] Hamamura K. . Attenuation of malignant phenotypes of breast cancer cells through eIF2α-mediated downregulation of Rac1 signaling. Int. J. Oncology 44, 1980–1988 (2014). 10.3892/ijo.2014.2366 24691491

[b19] Xu W. . Suppressed invasive and migratory behaviors of SW1353 chondrosarcoma cells through the regulation of Src, Rac1 GTPase, and MMP13. Cellular Signaling 27, 2332–2342 (2015). 10.1016/j.cellsig.2015.08.014 26303573

[b20] Parri M. & Chiarugi P. Rac and Rho GTPases in cancer cell motility control. Cell Commun Signal 8, 23 (2010). 20822528 10.1186/1478-811X-8-23 PMC2941746

[b21] Hamamura K. . *In vitro* and *in silico* analysis of an inhibitory mechanism of osteoclastogenesis by salubrinal and guanabenz. Cellular Signaling 27, 353–362 (2015). 10.1016/j.cellsig.2014.11.020 25435425

[b22] Coleman R. E. . Breast-cancer adjuvant therapy with zoledronic acid. N Engl J Med. 365, 1396–1405 (2011). 21995387 10.1056/NEJMoa1105195

[b23] Smith M. R. . Denosumab and bone-metastasis-free survival in men with castration-resistant prostate cancer: results of a phase 3, randomised, placebo-controlled trial. Lancet 379, 39–46 (2012). 22093187 10.1016/S0140-6736(11)61226-9 PMC3671878

[b24] Durie B. G., Katz M. & Crowley J. Osteonecrosis of the jaw and bisphosphonates. N Engl J Med. 353, 99–102 (2005). 10.1056/NEJM200507073530120 16000365

[b25] Sasaki A. . Bisphosphonate risedronate reduces metastatic human breast cancer burden in bone in nude mice. Cancer Res 55, 3551–3557 (1995). 7627963

[b26] Wang H. . The osteogenic niche promotes early-stage bone colonization of disseminated breast cancer cells. Cancer Cell. 27, 193–210 (2015). 25600338 10.1016/j.ccell.2014.11.017 PMC4326554

[b27] Borcherding D. C. . Expression and therapeutic targeting of dopamine receptor-1 (D1R) in breast cancer. Oncogene 35, 3103–3113 (2015). 26477316 10.1038/onc.2015.369 PMC5541367

[b28] Jandaghi P. . Expression of DRD2 is increased in human pancreatic ductal adenocarcinoma and inhibitors slow tumor growth in mice. Gastroenterology 151, 1218–1231 (2016). 27578530 10.1053/j.gastro.2016.08.040

[b29] Malochet-Guinamand S., Durif F. & Thomas T. Parkinson’s disease: a risk factor for osteoporosis. Joint Bone Spine 82, 406–410 (2015). 26453100 10.1016/j.jbspin.2015.03.009

[b30] Lelekakis M. . A novel orthotopic model of breast cancer metastasis to bone. Clinical Experimental Metastsis 17, 163–170 (1999). 10.1023/a:1006689719505 10411109

[b31] Cailleau R., Young R., Olivé M. & Reeves W. J. Jr. Breast tumor cell lines from pleural effusions. J Natl Cancer Inst. 53, 661–674 (1974). 4412247 10.1093/jnci/53.3.661 PMC7364228

[b32] Cailleau R., Olivé M. & Cruciger Q. V. Long-term human breast carcinoma cell lines of metastatic origin: preliminary characterization. In Vitro. 14, 911–915 (1978). 730202 10.1007/BF02616120

[b33] Soule H. D. & McGrath C. M. A simplified method for passage and long-term growth of human mammary epithelial cells. In Vitro Cell Dev Biol. 22, 6–12 (1986). 2418007 10.1007/BF02623435

[b34] Kebabian J. W. . A-77636: a potent and selective dopamine D1 receptor agonist with antiparkinsonian activity in marmosets. Eur J Pharmacol 229, 203–209 (1992). 1362704 10.1016/0014-2999(92)90556-j

[b35] Kamimura Y. . Differential enhancing effects of alpha2,8-sialyltransferase on the cell proliferation and mobility. Int J Oncol. 26, 337–344 (2005). 15645117

[b36] Bunt G. & Wouters F. S. Visualization of molecular activities inside living cells with fluorescent labels. Int Rev Cytol. 237, 205–277 (2004). 15380669 10.1016/S0074-7696(04)37005-1

[b37] Raschke W. C., Baird S., Ralph P. & Nakoinz I. Functional macrophage cell lines transformed by Abelson leukemia virus. Cell. 15, 261–267 (1978). 212198 10.1016/0092-8674(78)90101-0

[b38] Sun W., Shi Y., Lee W. C., Lee S. Y. & Long F. Rictor is required for optimal bone accrual in response to anti-sclerostin therapy in the mouse. Bone 85, 1–8 (2016). 26780446 10.1016/j.bone.2016.01.013 PMC4896354

[b39] Connolly E. M. . Cyclo-oxygenase inhibition reduces tumour growth and metastasis in an orthotopic model of breast cancer. Br J Cancer 87, 231–237 (2002). 12107848 10.1038/sj.bjc.6600462 PMC2376100

[b40] Teixeira A. . Novel method to quantify traction in a vitrectomy procedure. Br J Ophthalmol 94, 1226–1229 (2010). 20538657 10.1136/bjo.2009.166637

[b41] Mabilleau G. . Comparison between quantitative X-ray imaging, dual energy X-ray absorptiometry and microCT in the assessment of bone mineral density in disuse-induced bone loss. J Musculoskelet Neuronal Interact 15, 42–52 (2015). 25730651 PMC5123607

[b42] Susan M. Ott. Histomorphometric Measurements of Bone Turnover, Mineralization, and Volume. Clin J Am Soc Nephrol. 3, S151–S156 (2008). 18988700 10.2215/CJN.04301206 PMC3152285

[b43] Parfitt A. M. . Bone histomorphometry: standardization of nomenclature, symbols, and units. Report of the ASBMR histomorphometry nomenclature committee. J Bone Miner Res 2, 595–610 (1987). 3455637 10.1002/jbmr.5650020617

