# Author Correction: Inhibitory effects of dopamine receptor D_1_ agonist on mammary tumor and bone metastasis

**DOI:** 10.1038/s41598-022-22386-8

**Published:** 2022-11-03

**Authors:** Kazumasa Minami, Shengzhi Liu, Yang Liu, Andy Chen, Qiaoqiao Wan, Sungsoo Na, Bai-Yan Li, Nariaki Matsuura, Masahiko Koizumi, Yukun Yin, Liangying Gan, Aihua Xu, Jiliang Li, Harikrishna Nakshatri, Hiroki Yokota

**Affiliations:** 1 Department of Biomedical Engineering, Indiana University Purdue University Indianapolis, Indianapolis, IN 46202 USA; 2 Department of Medical Physics & Engineering, Osaka University Graduate School of Medicine Suita, Osaka, 565-0871 Japan; 3 Department of Pharmacology, School of Pharmacy, Harbin Medical University, Harbin, 150081 China; 4 Weldon School of Biomedical Engineering, Purdue University, West Lafayette, IN 47907 USA; 5 Osaka Medical Center for Cancer and Cardiovascular Diseases, Osaka, 537-8511 Japan; 6 Department of Biology, Indiana University Purdue University Indianapolis, Indianapolis, IN 46202 USA; 7 Department of Surgery, Simon Cancer Research Center, Indiana University School of Medicine, Indianapolis, IN 46202 USA

Correction to: *Scientific Reports* 10.1038/srep45686, published online 04 April 2017

This Article contains an error in Figure 2D where the image for DMSO control siRNA at 0 h was inadvertently duplicated from the image for DMSO control at 0 h.

In addition, in the Supplementary Figure 2E, the images for eIF2α were incorrectly taken from the images for non-specifically stained band images, and in the legend micromolar “µM” was incorrectly given twice as millimolar “mM”.

The correct Figure 2 and Supplementary Figure 2 and accompanying legend appear below.

**Figure 2:**
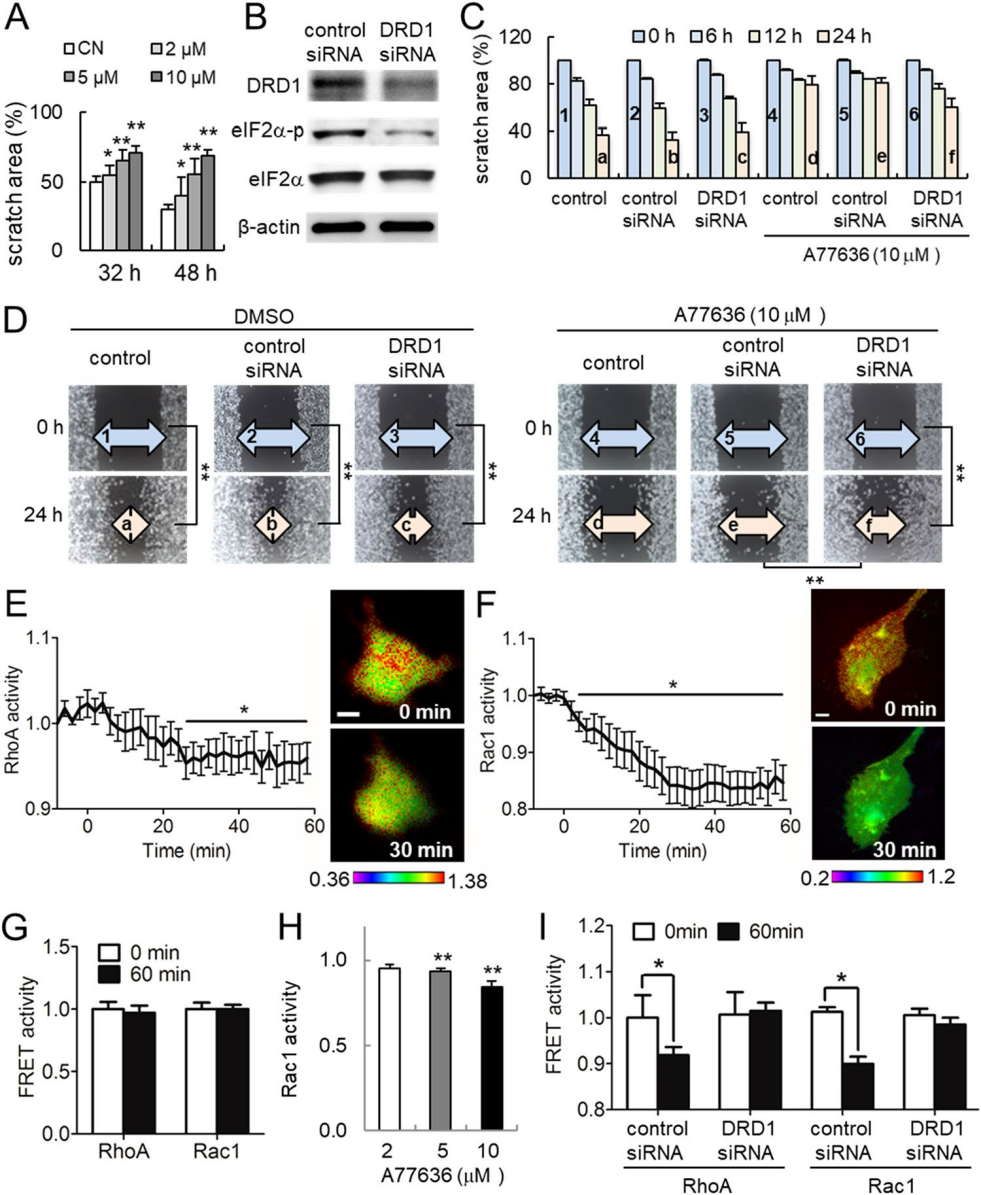
Inhibitory effects of A77636 on the migration of MD231 cells. The single and double asterisks indicate *p* < 0.05 and *p* < 0.01, respectively. (**A**) Dose-dependent suppression of cellular migration in response to 2, 5, and 10 μM A77636. Of note, CN = control. (**B**) Partial silencing of dopamine receptor D1 (DRD1) with DRD1 siRNA. (**C**, **D**) Wounded area with and without DRD1 siRNA in the presence and absence of 10 μM A77636. The double asterisk indicates *p* < 0.01. (**E**, **F**) FRET-based activities of RhoA and Rac1 GTPases, respectively, in response to 10 μM A77636. (**G**) Stationary FRET-based baseline activities of RhoA and Rac1 GTPases in the absence of A77636. (**H**) Rac1 activity after 1 h treatment with 1, 5, and 10 μM A77636. (**I**) FRET-based RhoA and Rac1 activities in the presence and absence of DRD1 siRNA in response to 10 μM A77636.

## Supplementary Information

Supplementary Information.

