# Supplementary material for: Inhibitory Effects of Dopamine Receptor D1 Agonist on Mammary Tumor and Bone Metastasis
Source: Sci Rep. 2017 Apr 4;7:45686. doi: 10.1038/srep45686 (PMC5379485; doi:10.1038/srep45686)
Supplement: Supplementary Information [file srep45686-s1.pdf]

Supplementary Information

Inhibitory Effects of Dopamine Receptor D_1 Agonist on Mammary Tumor and Bone Metastasis

Kazumasa Minami[1,2], Shengzhi Liu[1,3], Yang Liu[1,3], Andy Chen[4], Qiaoqiao Wan[1], Sungsoo Na[1], Bai-Yan Li[3], Nariaki Matsuura[5], Masahiko Koizumi[2], Yukun Yin[6], Liangying Gan[6], Aihua Xu[6], Jiliang Li[6], Harikrishna Nakshatri[7], Hiroki Yokota[1]

[1]Department of Biomedical Engineering, Indiana University Purdue University Indianapolis, Indianapolis, IN 46202, USA
[2] Department of Medical Physics & Engineering Osaka University Graduate School of Medicine Suita, Osaka 565-0871, Japan
[3]Department of Pharmacology, School of Pharmacy, Harbin Medical University, Harbin 150081, China
[4]Weldon School of Biomedical Engineering, Purdue University, West Lafayette, IN 47907, USA
[5]Osaka Medical Center for Cancer and Cardiovascular Diseases, Osaka 537-8511, Japan
[6]Department of Biology, Indiana University Purdue University Indianapolis, Indianapolis, IN 46202, USA
[7]Department of Surgery, Simon Cancer Research Center, Indiana University School of Medicine, Indianapolis, IN 46202, USA

Methods

Two-staged MTT screening. In the first-stage screening, an MTT assay was conducted for 10 mM of each of 1,120 biologically active compounds in a Tocriscreen library (Tocris Bioscience, Ellisville, MO, USA) using MDA-MB-231 cells (American Type Culture Collection - ATCC, Manassas, VA, USA). Cells (2 x 10^3/well) were seeded in 96-well plates in the medium consisting of 1% FBS. After 3 day incubation with one of the compounds, the reduction of MTT to formazan was evaluated by measuring the absorbance at 570 nm with a plate reader (EL800, BioTek, Winooski, VT, USA). We selected 50 best compounds that lowered the MTT absorbance. In the second-stage screening, an MTT assay was conducted with three other cell lines including MDA-MB-436 breast cancer cells (ATCC), MCF10A breast epithelial cells (ATCC), and MC3T3 osteoblast-like cells (Sigma-Aldrich, St. Louis, MO, USA).

Cluster analysis and selection criteria for drug candidates. Using the second-stage screening data, *k*-means clustering was applied in R (v 3.1.0) to the MTT survival rates in the 4 cell lines to find similarly behaving drugs. Additionally, a performance index was calculated by subtracting the sum of the survival rates of cancer cell lines (MDA-MB-231 and MDA-MB-436) by the sum of the survival rates of the normal tissue cell lines (MCF10A and MC3T3). Drug candidates were chosen by considering the performance index, the types of drugs in each cluster, and the survival of the MC3T3 cells.

X-ray imaging and clinical score. Using X-ray images of the right hindlimbs, the clinical scores were defined from 0 to 3: “0” for no sign of osteolytic lesion, “1” for minor osteolytic lesion, “2” for moderate osteolytic lesion, and “3” for major osteolytic legion. Four independent scorers participated in the evaluation.

Bone degradation ratio and tumor area ratio. Using histological sections of the distal femur from the placebo and A77636-treated groups, the bone degradation ratio was defined as: (length of degraded/missing parts of the cortical bone) divided by (total length of the cortical bone). The tumor area ratio was calculated as: (tumor area) divided by (cross-sectional area of all tissue).

Determination of bone mineral density (BMD). The tibia, femur, humerus and ulna were isolated, and the BMD were measured using LUNAR Piximus.

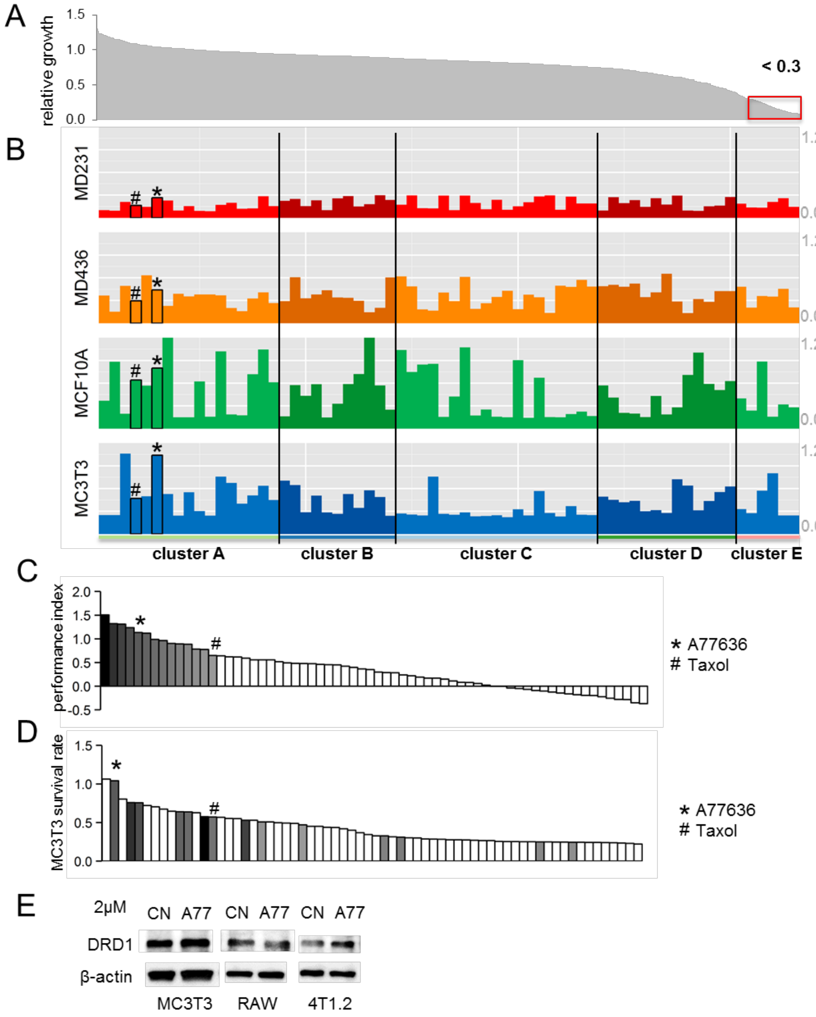

Supplementary Figure 1. Selection of A77636 as a potential drug candidate. (A) Relative growth of MD231 cells in response to each of 1,120 biologically active compounds. Sixty-six agents in the far right end with the normalized growth < 0.3 were selected for further screening. (B) Bar charts, illustrating the relative growth of 4 cell lines (MD231, MD436, MCF10A, and MC3T3) in response to 63 selected compounds in (A). MD231 and MD436 are breast cancer cell lines, while MCF10A and MC3T3 are non-cancerous epithelial and osteoblast like cell lines, respectively. (C) Sixty-three top performing candidates with their performance index, which is a linear combination of inhibition rates for 2 cancer cell lines and survival rates for 2 normal cell lines. Of note, * and # denote A77636 and Taxol, respectively. (D) Survival rate of MC3T3 osteoblast-like cells in the presence of 63 selected agents. A77636 and Taxol are ranked 2nd and 25th for maintaining MC3T3 cells. (E) Expression of dopamine receptor D1 (DRD1) protein in three mouse cell lines (MC3T3, RAW264.7, and 4T1.2) used in this study. Cells were treated with 2 μM A77636 (A77) for 6 h.

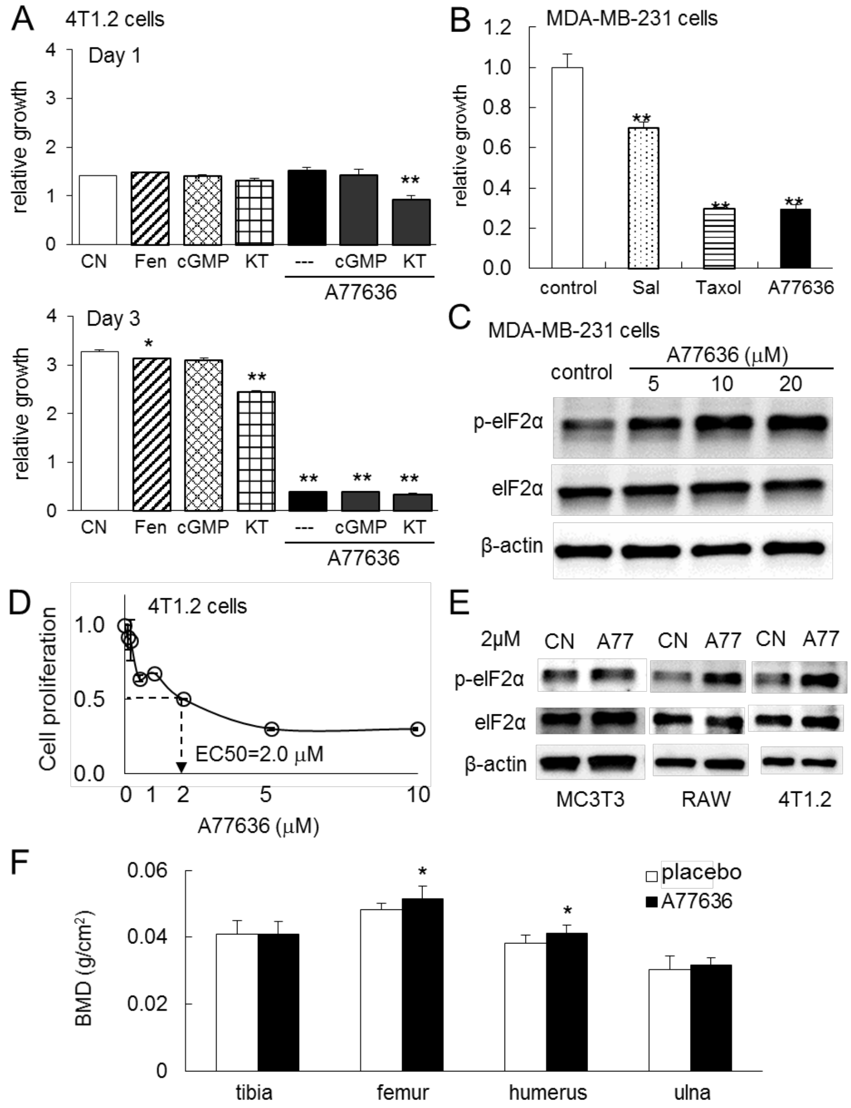

Supplementary Figure 2. A77636's effect on 4T1.2 cells, MD231 cells, and bone mineral density (BMD) of 4 long bones in the mouse model of mammary tumor. The single and double asterisks indicate $p < 0.05$ and $p < 0.01$, respectively. (A) Relative growth of 4T1.2 cells in response to 10 mM Fenoldopam (Fen; selective DRD1 partial agonist), cGMP analog (cGMP), KT5823 (KT; inhibitor of protein kinase G), A77636, A77636 with cGMP analog, and A77636 with KT5823. (B) Relative growth of MD231 cells in response to10 mM salubrinal (Sal), Taxol, and A77636. (C) Upregulation of eIF2a phosphorylation by A77636 in MD231 cells. (D) Estimation of EC50% for blocking 50% of cell proliferation in 4T1.2 cells for 2 days. (E) Expression of phosphorylated eIF2α (p-eIF2α) in response to 2 μM A77636 for 6 h in three cell lines (MC3T3, RAW264.7, and 4T1.2). Of note, A77 = A77636, and CN = control. (F) BMD of the tibia, femur, humerus, and ulna in the mouse model of mammary tumor.

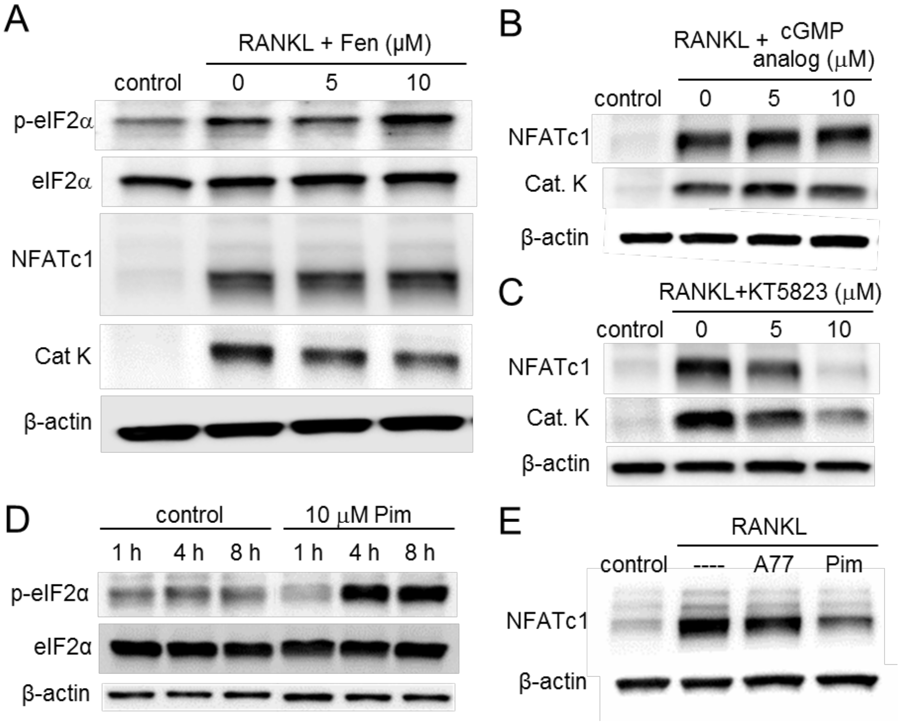

Supplementary Figure 3. Effects of Fenoldopam, cGMP analog, KT5823, and Pimozide on development of RAW264.7 pre-osteoclast cells. (A) Effects of Fenoldopam on p-eIF2α, NFATc1, and Cathepsin K. (B&C) Effects of cGMP analog and KT5823 on expression of NFATc1 and cathepsin K with cGMP analog and KT5823, respectively. (D) Elevation of p-eIF2α by 10 μM Pimozide (Pim). (E) Downregulation of NFATc1 by 10 μM A77636 and 10 μM Pimozide.

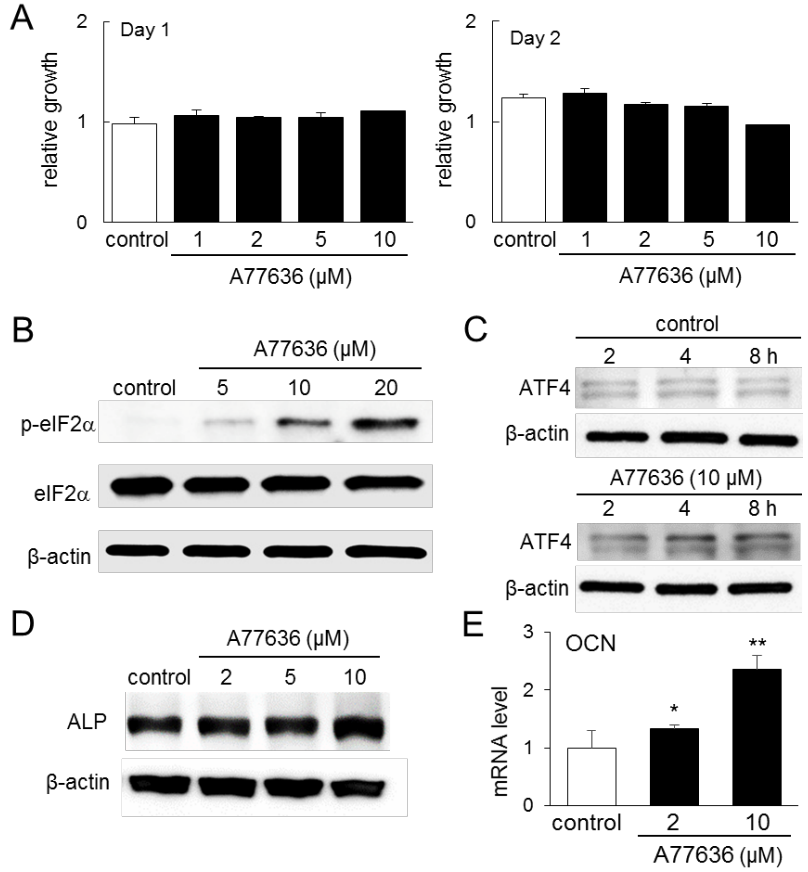

Supplementary Figure 4. Stimulatory effects of A77636 on development of MC3T3 osteoblast-like cells. (A) Relative cell growth of MC3T3 cells in response to 1, 2, 5, and 10 μM A77636 on days 1 and 2. (B) Dose dependent elevation of the phosphorylation level of eIF2α in response to 5, 10, and 20 μM A77636. (C) Elevation of ATF4 protein in response to 10 μM A77636. (D) Expression of alkaline phosphatase (ALP) in response to A77636. (E) Dose-dependent elevation of osteocalcin (OCN) mRNA in response to 2 and 5 μM A77636 for 6 days. The single and double asterisks indicate $p < 0.05$ and $p < 0.01$, respectively.

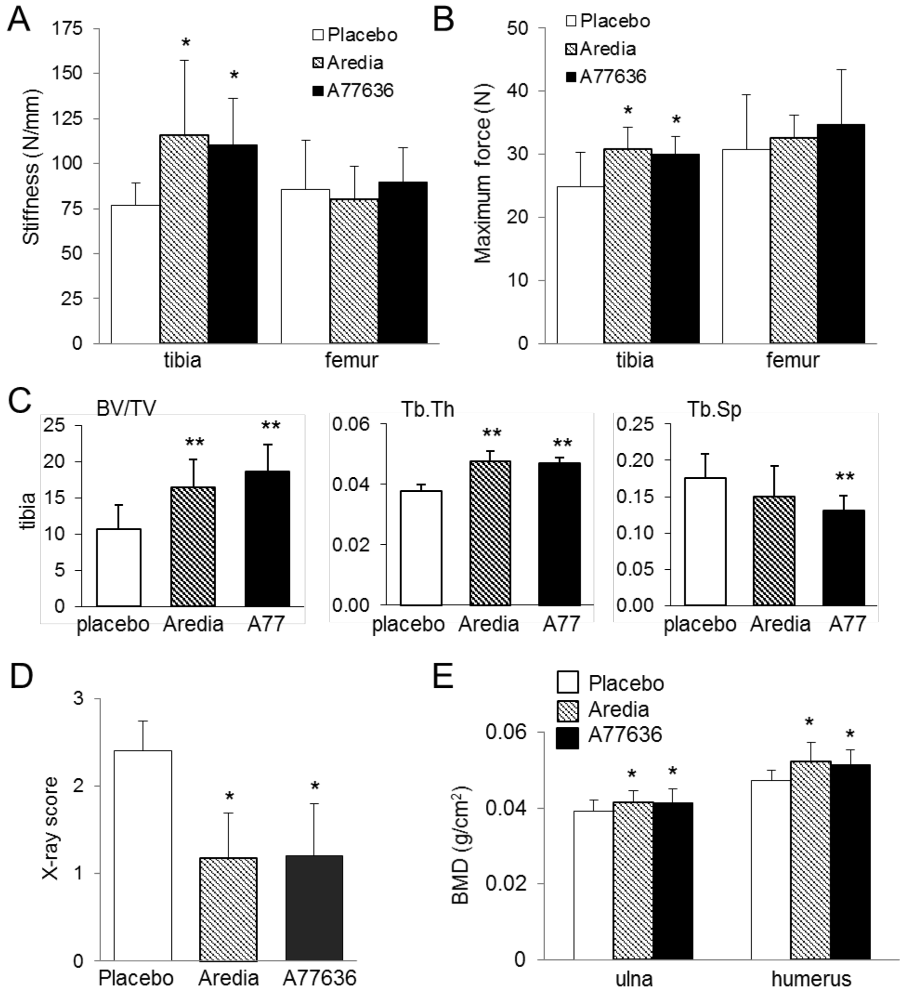

Supplementary Figure 5. A77636-driven protection of bone in the mouse model of bone metastasis. The single and double asterisks indicate $p < 0.05$ and $p < 0.01$, respectively. (A&B) Stiffness (N/mm) and maximum force (N) of the tibia and femur in the placebo control, Aredia-treated, and A77636-treated groups. (C) Three parameters for trabecular bone in the proximal tibia. Of note, A77 = A77636, BV/TV = bone volume normalized by tissue volume, Tb.Th = trabecular thickness, and Tb.Sp = trabecular spacing. (D) X-ray score of the right femur. Of note, score 3 = severe osteolysis, 2 = mild osteolysis, 3 = minor osteolysis, and 0 = no apparent osteolysis. (E) Bone mineral density (BMD) of the ulna and humerus.

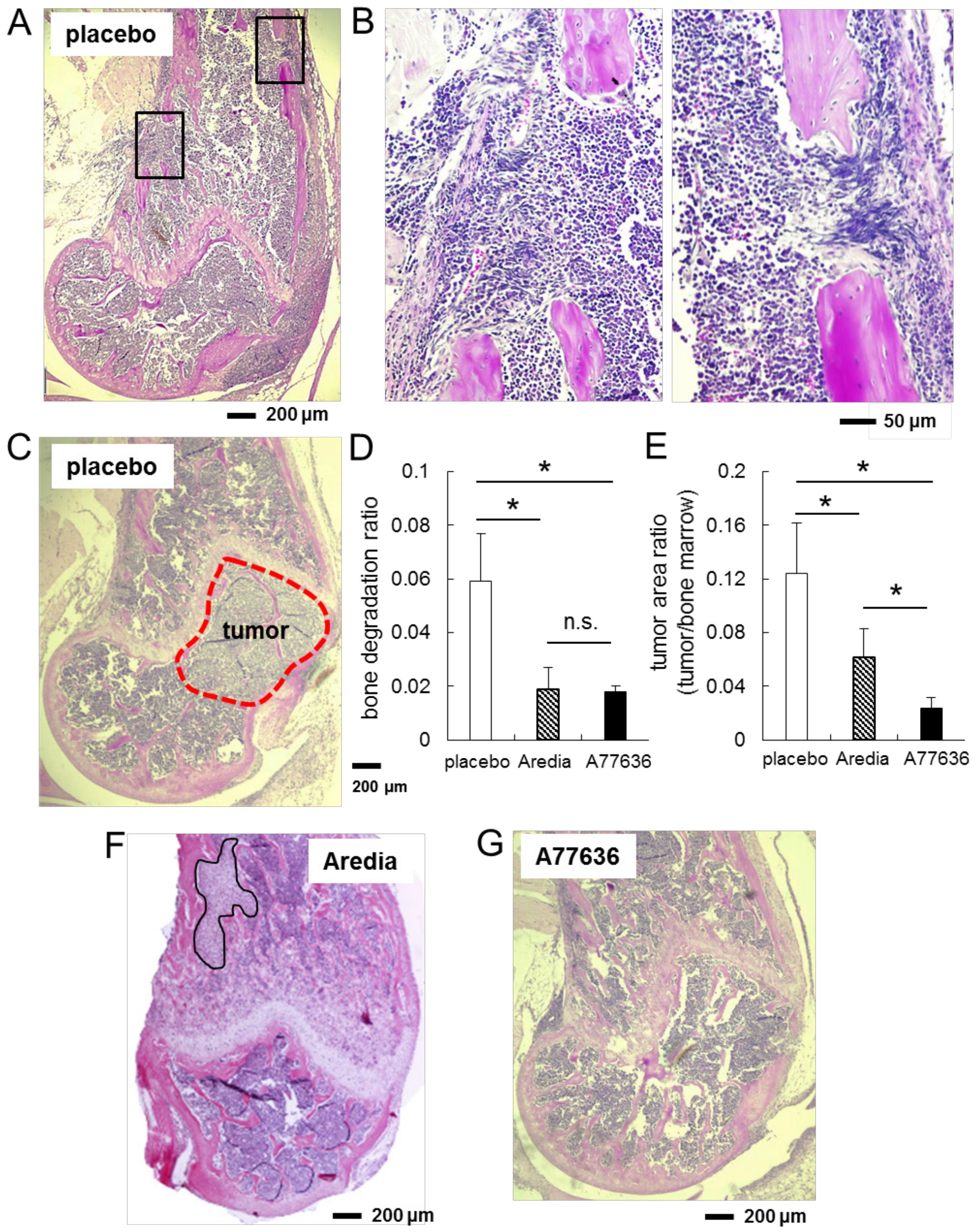

Supplementary Figure 6. Histological analysis of bone metastasis. The single asterisk indicates $p < 0.05$. (A-C) Representative images of the distal femur in the placebo group. The boxes in (A) are enlarged in (B), which presents osteolytic cortical bone in the distal femur. The area, surrounded by the dotted curve in (C), indicates a cluster of tumor cells. (D&E) Comparison of bone degradation ratio and tumor area ratio, respectively, among the placebo, Aredia-treated and A77636-treated groups. Of note, n.s. = not significant. (F) Aredia-treated distal femur. The area, surrounded by the dotted curve, indicates a cluster of tumor cells. (G) A77636-treated distal femur.
